# T200. DISTINCT ASSOCIATIONS OF MOTOR DOMAINS WITH THE GENETIC RISK FOR PSYCHOSIS – DIFFERENT PATHWAYS TO MOTOR ABNORMALITIES IN SCHIZOPHRENIA?

**DOI:** 10.1093/schbul/sby016.476

**Published:** 2018-04-01

**Authors:** Lea Schäppi, Katharina Stegmayer, Petra Viher, Sebastian Walther

**Affiliations:** University Hospital of Psychiatry

## Abstract

**Background:**

Aberrant motor function is an integral part of Schizophrenia. In fact, abnormalities are frequently found in patients, in populations at risk, and in unaffected relatives. Motor abnormalities are suspected to be relevant for the clinical outcome and could probably predict the conversion from at-risk individuals to schizophrenia. Furthermore, motor function and has been argued as endophenotype of the disorder. Yet, which particular motor domain may classify as a potential endophenotype is unknown. We aimed to compare schizophrenia patients, unaffected first degree relatives and healthy controls for different motor domains. We expected impairments in all domains in patients and in some domains in relatives.

**Methods:**

We included 43 schizophrenia patients, 34 unaffected first degree relatives of schizophrenia patients and 29 healthy control subjects, matched for age, gender and education level. We compared motor function of five domains between the groups. The domains comprise neurological soft sings (NSS), abnormal involuntary movements (dyskinesia), Parkinsonism, complex fine motor function applying the coin rotation task as well as finger tapping. Furthermore, we tested the association of motor function of the five domains with working memory, frontal lobe function and nonverbal intelligence for each group separately using within-group bivariate correlations.

**Results:**

Schizophrenia patients showed poorer motor function in all tested domains compared to healthy controls. First-degree relatives had intermediate ratings with aberrant function in two motor domains. In detail, relatives had significantly more NSS and performed poorer in the finger tapping task than controls. In contrast, in relatives complex fine motor function was intact. Relatives did not differ from controls in dyskinesia or Parkinsonism severity.

**Discussion:**

Taken together, schizophrenia patients have motor abnormalities in all tested domains. Thus, motor abnormalities are a key element of the disorder. Likewise, first degree relatives presented motor deficits in two domains. A clear difference between relatives and healthy controls was found for NSS and finger tapping. Thus, NSS and finger tapping may be a potential marker of vulnerability for schizophrenia. The lack of association between genetic risk and dyskinesia or Parkinsonism suggests distinct pathobiological mechanisms in the various motor abnormalities in schizophrenia.

